# Is There a Potential of Misuse for Venlafaxine and Bupropion?

**DOI:** 10.3389/fphar.2018.00239

**Published:** 2018-03-21

**Authors:** Fabrizio Schifano, Stefania Chiappini

**Affiliations:** Psychopharmacology, Drug Misuse, and Novel Psychoactive Substances Research Unit, School of Life and Medical Sciences, University of Hertfordshire, Hatfield, United Kingdom

**Keywords:** antidepressant misuse, bupropion abuse, venlafaxine dependence, venlafaxine withdrawal, EMA, Yellow Card Scheme, paroxetine, fluoxetine

## Abstract

**Objective:** Traditionally, studies on the non-medical use of pharmaceutical products have focused on controlled substances; e.g., opiates/opioids; and benzodiazepines. Although both bupropion and venlafaxine have been reported as being misused, only anecdotal reports have been made available so far. Hence, the European Monitoring Agency (EMA) Adverse Drug Reactions (ADRs), misuse/abuse/dependence and withdrawal, venlafaxine- and bupropion-related, database was here analyzed.

**Methods:** All EMA spontaneous reports relating to venlafaxine (2005–2016) and bupropion (2003–2016) notifications were here analyzed, to provide a descriptive analysis by source, gender, age, and type of report. The UK-based, 2000–2016, Yellow Card Scheme pharmacovigilance database, bupropion and venlafaxine withdrawal reports were compared as well with those pertaining to fluoxetine and paroxetine.

**Results:** Out of 20,720 (bupropion) and 47,516 (venlafaxine) total number of ADRs, some 2,232 (10.8%), and 4,071 (8.5%) misuse/abuse/dependence ADRs were respectively associated with bupropion and venlafaxine. Conversely, bupropion withdrawal-related ADRs were here reported in 299/20,720 (1.44%) cases and in 914/47,516 (1.92%) cases for venlafaxine. Overall, all bupropion and venlafaxine misuse-/abuse-/dependence- and withdrawal-ADRs were related to a respective number of 264 and 447 patients. According to the Proportional Reporting Ratio (PRR) computation, in comparison with venlafaxine bupropion resulted to be more frequently misused/abused (PRR: 1.50), but less frequently associated with both dependence (PRR: 0.92) and withdrawal (PRR: 0.77) issues. Yellow Card Scheme data suggested that paroxetine and venlafaxine, in comparison with fluoxetine and bupropion, were associated with higher number of withdrawal-related reports.

**Conclusions:** The dopaminergic, stimulant-like, bupropion activities may be associated with its possible recreational value. Present data may confirm that the occurrence of a withdrawal syndrome may be a significant issue for venlafaxine-treated patients.

## Introduction

The misuse or abuse of prescription drugs is a recently emerging issue, becoming a reason of public concern (Schifano et al., 2015). Intentional misuse of prescribing medications involves gabapentinoids (Chiappini and Schifano, 2016); anticholinergics (Schifano and di Costanzo, 1991); a range of stimulants (Schifano et al., 2015); performance- and image-enhancing drugs (Schifano et al., 2015); and a few antipsychotics (Chiappini and Schifano, 2018). Although a worldwide rise of antidepressant (ADs) consumption (Kantor et al., 2015; OECD Indicators, 2015) has been described, there are only a few studies relating to the AD potential of misuse and withdrawal. The selective serotonin reuptake inhibitors/SSRIs have already been reported to be associated with a risk of both dependence and an early and late onset (Fava et al., 2015) occurrence of withdrawal syndrome (Chouinard and Chouinard, 2015; Cosci et al., 2015). However, according to an updated (July 2017) PubMed/Medline/Embase literature review here carried out, a few further ADs, e.g., bupropion and venlafaxine, have emerged as increasingly being misused (Evans and Sullivan, 2014; Schifano et al., 2015; Anderson et al., 2017); the following represents an overview of most significant related findings.

### Bupropion and venlafaxine; overview of clinical pharmacological and misusing issues

*Bupropion* is a second-generation AD that acts as a selective inhibitor of catecholamines' (noradrenaline and dopamine) reuptake, devoid of any serotonergic; antihistamine; or anticholinergic properties (Stahl et al., 2004). Furthermore, bupropion is a non-competitive antagonist of nicotinic acetylcholine receptors (Guzman, 2018), hence being prescribed for both major depressive episodes and as an aid in smoking cessation (EMA, 2003). Bupropion is also used ‘off label’ in a range of conditions, including: attention deficit/hyperactivity disorder, chronic fatigue, sexual dysfunction, and obesity. Bupropion adverse events typically include dry mouth, nausea, and insomnia.

Known as “welbys,” “wellies,” “dubs,” or “barnies,” its recreational use by oral or nasal routes was first described some 15 years ago (McCormick, 2002; Welsh and Doyon, 2002; GSK, 2016). More recently, reports of high-dose bupropion injecting have appeared as well (Baribeau and Araki, 2013), with people misusing the drug to get a “high” similar to the one obtained through other stimulants, such as cocaine. Adverse effects range from nasal pain to irritability, agitation, cardiac toxicity, hallucinations and seizures (Rettew and Hudziak, 2006; Stall et al., 2014). Schifano et al. (2015) analyzing specialized web fora posts related to the potential misuse/non-medical use of bupropion compared with amitriptyline and venlafaxine, identified a total of 7,756 references to at least one of them, and 668 (8.61%) of them referencing misuse or non-medical use of one of the three ADs, with bupropion accounting for 438 (65.6%). The most commonly reported desired effects were similar to stimulants with bupropion; sedative with amitriptyline; and dissociative with venlafaxine. The nasal route of administration was most frequently reported for bupropion, whereas the oral route was most frequently reported for amitriptyline and venlafaxine. Stassinos et al. (Stassinos and Klein-Schwartz, 2016) carried out a 14-year retrospective review on bupropion cases of intentional abuse reported to the US National Poison Data System, and identified 975 cases, with a prevalence increasing by 75%, from 2000 to 2012. Most cases were 13 to 29 years old (67.4%), with typical clinical effects being tachycardia (57.0%), seizures (33.5%), agitation/irritability (20.2%), hallucinations/delusions (14.0%), and tremor (13.1%). Most exposures were ingestions (745) followed by insufflation (166). Final management sites were predominantly emergency department (36.9%) and admission to critical care unit (27.3%) according to Toxnet (Toxnet, Toxicology Data Network, 2015). In line with this, a 2004–2011 search on the US Drug Abuse Warning Network (DAWN) system identified 210 cases of possible bupropion misuse and abuse, and in a minority of cases the molecule resulted to be snorted or injected (GSK, 2016).

Bupropion is a cathinone derivative (Lewin et al., 2014); similar to remaining molecules of this class, one could argue that its diversion potential and reinforcement of misuse may be related to its dopaminergic and noradrenergic effects (Vento et al., 2013). Most bupropion abusers present with a history of drug addiction (Khurshid and Decker, 2004; Hill et al., 2007; Langguth et al., 2009; Kim and Steinhart, 2010; Reeves and Ladner, 2013; Yoon and Westermeyer, 2013); higher prevalence levels have been identified in inmates, and this has brought to bupropion removal of from some US prison formularies (Laird and Narayan, 2009; Phillips, 2012; Hilliard et al., 2013).

*Venlafaxine* is indicated (HSCIC, 2016) for the treatment of major depressive episodes, generalized anxiety disorder and social phobia, with off-label uses including obsessive-compulsive disorder, and chronic pain syndromes (EMA, 2007). It is available both as an immediate and extended-release (XR) formulation; XR pills contain spherules in coated or encapsulated form which, when broken, release the medication rapidly (Muth et al., 1986).

Venlafaxine is a phenylethylamine derivative inhibiting the reuptake of serotonin/5-HT; norepinephrine/NE; and to a lesser extent dopamine/DA (Bolden-Watson and Richelson, 1993; Redrobe et al., 1998). The reuptake effects of venlafaxine are dose-dependent, with action on 5-HT transmission at low doses (<150 mg/day); on both 5-HT and NE systems at moderate doses (>150 mg/day); and on DA at high doses (>300 mg/day) (Harvey et al., 2000; Stahl, 2013). In the prefrontal cortex nerve terminals there are only few DA, but plenty of both NE and 5-HT, transporters (Weikop et al., 2004). Hence, if DA is released, it is free to circulate from the synapses, with huge prefrontal cortex levels of DA increase being recorded (Shang et al., 2007). Venlafaxine main active metabolite, desvenlafaxine, presents with large levels of NE transporter inhibitory activities, further increasing levels of DA turnover in the prefrontal cortex (Shang et al., 2007). Both venlafaxine and its metabolite do not possess any monoamine oxidase inhibitory activity, which is responsible for the degradation of DA (Maj and Rogóz, 1999; Shang et al., 2007). Preclinical studies showed that venlafaxine presents with a high affinity for D2 receptors (Bolden-Watson and Richelson, 1993; Shang et al., 2007), whilst its chronic administration is associated as well with D3 receptors' adaptive changes. Finally, venlafaxine desensitizes both 5-HT1A and beta adrenergic receptors (Maj and Rogóz, 1999), but virtually no affinity has been demonstrated for opiate; benzodiazepine; phencyclidine; N-methyl-D-aspartate; muscarinic; α_1_-adrenergic; or histaminergic receptors. Venlafaxine abrupt discontinuation may be associated with a withdrawal syndrome (Rudolph and Derivan, 1996; Augustin et al., 1997; Parker and Blennerhassett, 1998; Campagne, 2005; Taylor et al., 2005; Sabljić et al., 2011; Carvalho et al., 2016), characterized by nausea, depression, suicidal thoughts, disorientation, stomach cramps, panic attacks, sexual dysfunction, headache, and occasional psychotic symptoms (Koga et al., 2008); in some cases, the clinical picture may resemble a stroke (Campagne, 2005). Although how the withdrawal syndrome develops is unknown, it may well be associated with electrophysiological changes in 5-HT receptors. This is similar to what can be observed with the SSRIs, although the severity of withdrawal may be higher with venlafaxine (Fava et al., 1997). SSRIs and SNRIs have replaced the habit-forming benzodiazepines for the treatment of anxiety (Cosci et al., 2015), however dependence and withdrawal symptoms associated with newer ADs have been described (Sir et al., 2005; Stahl et al., 2005; Llorca and Fernandez, 2007; Kotzalidis et al., 2008; Fava et al., 2015). Consistent with this, Kelly et al. (Kelly et al., 2014) observed that cannabis-dependent participants with depressive disorder are less likely to achieve abstinence when exposed to venlafaxine treatment, suggesting that withdrawal-like symptoms led to continued marijuana smoking in this group. Finally, since venlafaxine and its metabolites cross the placenta, even the newborn can be exposed to the risk of a discontinuation syndrome, which is at times associated with encephalopathy or paroxysmal episodes (Holland and Brown, 2017). Although venlafaxine withdrawal can successfully be managed with a short course of duloxetine (Cutler, 2017), in order to taper down gradually its dosage the use of tapering strips, containing a slightly lower dose of medication on each consecutive day, has been suggested (Groot, 2013). Apart from the occurrence of withdrawal, the intake of large venlafaxine (“baby ecstasy”) dosages has been reported (Francesconi et al., 2015). Related effects have anecdotally been described as amphetamine/ecstasy-like, with the mechanism behind its putative abuse liability arguably being related to venlafaxine increased dopaminergic turnover at high dosages (Shang et al., 2007). In line with this, using wastewater analysis, Baker et al. (Baker et al., 2014) presented community-wide estimation of drug and pharmaceuticals' consumption in England. Target compounds were compared to NHS prescription statistics; discrepancies were observed for a range of molecules, including venlafaxine, suggesting sales of non-prescribed venlafaxine. Furthermore, Fountain and Slaughter (Fountain and Slaughter, 2016) carried out a retrospective review of records of New Zealand National Poisons Centre records referring to the period 2003–2012; high or rapidly increasing levels of enquiries were identified for a range of prescribing medicines, including venlafaxine.

To better define the context for change in ADRs over time, it may be important to consider bupropion and venlafaxine prescription rates' historical information. Although, for a range of reasons, the worldwide prescription figures for these molecules are not available (Chiappini and Schifano, 2018), England-based data from the Prescription Cost Analysis (Data.gov.uk, 2016) were here taken into account as a reference, hence serving as a general indication of prescription trends. When considering the available January 2009-December 2016 time-frame, the number of venlafaxine prescription items dispended increased over time, from 2.51 (2009) to 3.9 million (2016). Conversely, the bupropion prescribing rates showed here an opposite trend, decreasing from 0.51 to 0.22 million, possibly because bupropion is licensed, in the UK, only as an adjunct in nicotine cessation programmes, and not as an antidepressant. Over the last few years, a few more nicotine dependence pharmacotherapies have been made available, with possible decreasing levels of interest toward bupropion prescribing.

To assess both venlafaxine and bupropion misuse/abuse/dependence and withdrawal-related issues, we aimed here at analyzing the European Medicines Agency (EMA) EudraVigilance (EV) database (Schifano and di Costanzo, 1991), which collects electronic reports of suspected adverse drug reactions (ADRs) for all medicinal products authorized in the European Economic Area/EEA.

## Methods

After a formal request to EMA, we were allowed to access and analyze the EV ADRs database, relating to all venlafaxine- and bupropion-related case reports submitted spontaneously up to mid-July 2016; for a thorough description of the EV database refer to Chiappini and Schifano (2016, 2018). In order to focus on misuse; abuse; dependence; and withdrawal issues, in the two datasets we selected and identified the following ADRs: “dependence,” “drug abuse,” “drug abuse(r),” “drug dependence,” “drug diversion,” “drug withdrawal headache,” “drug withdrawal syndrome,” “intentional product misuse,” “intentional product use issue,” “substance abuse,” “substance dependence,” and “withdrawal syndrome.” Specifically, “misuse” was here meant to be the “intentional and inappropriate use of a product other than as prescribed or not in accordance with the authorized product information.” Conversely, “abuse” was here defined as the “intentional non-therapeutic use of a product for a perceived reward or desired non-therapeutic effect including, but not limited to, getting high/euphoria” (MedDRA, 2012). The term “addiction,” typically replaced by “dependence,” is the “overwhelming desire to take a drug for non-therapeutic purposes together with the inability to control or stop its use despite harmful consequences” (MedDRA, 2012). Finally, “withdrawal” was here defined as: “a substance-specific syndrome which follows cessation or reduction in the intake of a psychoactive substance previously regularly used” (WHO Expert Committee on Addiction-Producing Drugs, 2016). Withdrawal is at times considered as an additional indication of misuse, together with abuse; and dependence-related terms. However, although withdrawal symptoms may be indicative of physical dependence to a range of abusing drugs, there may also be withdrawal symptoms that are not necessarily related to the addictive and abuse properties of drugs; this is the case, for example, of beta blockers (Hopper et al., 2014) and corticosteroids (Shenouda et al., 2018). Hence, data relating to withdrawal were presented separately from those pertaining to misuse; abuse and dependence. To better assess the venlafaxine and bupropion associated withdrawal issues, we carried out a further comparison with paroxetine and fluoxetine, two SSRIs being characterized by different levels of withdrawal presentation during tapering down regime (Wilson and Lader, 2015). In doing so, we took into account the January 2000-December 2016 Drug Analysis Profiles pharmacovigilance data (MHRA, 2018) available from the Yellow Card Scheme (Yellow Card, 2018) of the UK-Medicines and Healthcare products Regulatory Agency (MHRA). MHRA collect reports of ADRs reported from within the UK, and these reports are then consistently forwarded to EMA (EMA, 2013), hence formally contributing to the EV database implementation.

ADRs' numbers differed from those referring to case reports, since different reporters/senders could have independently flagged the same ADR to EMA. We included here those ADRs which were listed as the “suspect drug,” meaning that the reporter suspected this drug, and not the concomitant medicine(s), to have caused the index ADR (Chiappini and Schifano, 2018). To more properly assess current data, the proportional reporting ratio (PRR) approach was considered, here defined as: “the ratio between the frequency with which a specific adverse event is reported for the drug of interest (relative to all adverse events reported for the drug) and the frequency with which the same adverse event is reported for the drug(s) in the comparison group (relative to all adverse events for drugs in the comparison group; EMA, 2008). Being a measure of disproportionality, a PRR > 1 suggests that the adverse event is more commonly reported for individuals taking the drug of interest relative to the comparison drug(s). The PRR is computed with the help of the following formula:

$$\begin{array}{l} \frac{\text{W}/\text{W}+\text{X}}{\text{Y}/\text{Y}+\text{Z}} \end{array}$$

(where: W = number of bupropion cases relating to the chosen adverse event(s); X = number of bupropion cases involving any other adverse events; Y = number of venlafaxine cases relating to the chosen adverse event(s); and Z = number of venlafaxine cases involving any other adverse events).

### Ethics' issues

Because of EMA protection of privacy and integrity of individuals, certain data elements (e.g., names/identifiers of individuals involved; country specific information, nationally authorized products etc.) were here not disclosed. As a consequence of the database analyzed containing only de-identified data, and consistent with previous reports (Chiappini and Schifano, 2016), no specific ethical issues were here identified.

## Results

Out of 20,720 for bupropion, and 47,516 for venlafaxine, total number of ADRs, some 2,232 (10.8%), and 4,071 (8.5%) misuse/abuse/dependence ADRs were respectively associated with bupropion and venlafaxine. Conversely, bupropion withdrawal ADRs were here reported in 299/20,720 (1.44%) cases and in 914/47,516 (1.92%) cases for venlafaxine (see Tables 1, 2).

**Table 1:** Overview of data relating to bupropion and venlafaxine ADRs as reported to the EV database.

	**Bupropion ADRs**	**Venlafaxine ADRs**
Time-frame considered	01/2005–05/2016	06/2003–07/2016
Total number of ADRs	20,720	47,516
Misuse-/abuse-/dependence- and withdrawal- related ADRs	2,531 (including misuse-/abuse-/dependence-related ADRs 2,232 and withdrawal-related ADRs 299)	4,985 (including misuse-/abuse-/dependence-related ADRs 4,071 and withdrawal-related ADRs 914)
Number of unique patients being reported to the database	264	447
Age range most typically represented	18–64 yy (64.5%)	18–64 yy (61.48%)
ADRs most typically represented within the misuse-/abuse-/dependence- and withdrawal- related ADRs' group	Drug abuse (61.6%), drug dependence (26.6.0%), drug withdrawal syndrome (11.8%)	Drug abuse (47.4%), drug dependence (34.3%), drug withdrawal syndrome 18.3(%)
Gender most typically represented	Male (F/M ratio: 1,155/1,257 = 0.91)	Female (F/M ratio: 2,483/2,406 = 1.03)
Concomitant drugs most typically represented	Opiates/opioids (in *n* = 123/264; 46.5 %); other antidepressants (in *n* = 116/264; 43.9% of cases, with SSRIs-citalopram, escitalopram, fluoxetine, paroxetine and sertraline being those most typically reported); other psychotropic substances, such as amphetamine, caffeine, cannabis, cocaine, ethanol, nicotine (in *n* = 68/264; 25.7%)	Opiates/opioids (in *n* = 150/447,33.55% of cases); benzodiazepines (in *n* = 138/447; 30.8%); and other antidepressants (in *n* = 114/447; 25.5% with SSRIs being those most typically reported)

**Table 2:** Bupropion and venlafaxine misuse/abuse-; dependence-; withdrawal and remaining-related ADRs': occurrence and proportional reporting ratio (PRR).

**Bupropion ADRs**	**No. of reactions ADRs**	**Proportion of bupropion ADRs**	**Bupropion vs. venlafaxine PRR**
Misuse/abuse-related ADRs (A1)	1,558	0.075	1.50
Dependence–related ADRs (A2)	674	0.032	0.92 (reverse: 1.09)
Withdrawal-related ADRs (A3)	299	0.014	0.77 (reverse: 1.30)
Other Adverse Events (B)	18,189	0.878	
Total (A1+A2+A3 +B)	20,720	1	
**Venlafaxine ADRs**	**No. of reactions ADRs**	**Proportion of venlafaxine ADRs**	
Misuse/abuse-related ADRs (C1)	2,361	0.05	
Dependence–related ADRs (C2)	1,710	0.036	
Withdrawal syndrome-related ADRs (C3)	914	0.019	
Other adverse events (D)	42,531	0.895	
Total (C1+C2+C3+D)	47,516	1	

The total number of ADRs corresponded to 264 and 447 patients respectively prescribed with bupropion (Jan 2005-May 2016) and venlafaxine (June 2003-July 2016).

**Figure 1:**
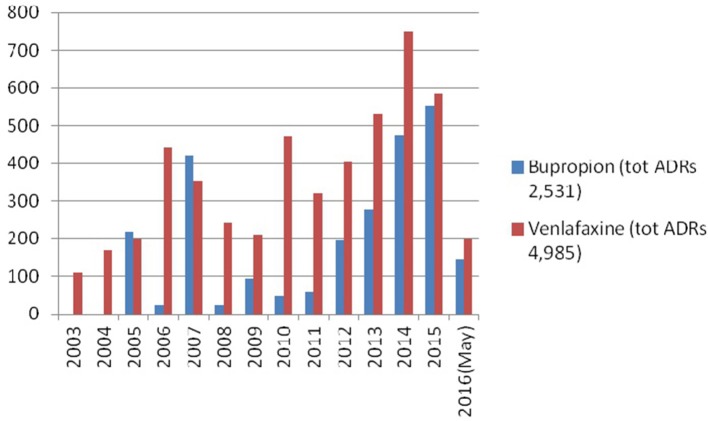
Data related to ADRs rates by year.

Over time, both bupropion and venlafaxine reports were on the increase (Figure 1); bupropion-related ADRs increased from a number of 48 in 2010 to 553 in 2015. Venlafaxine-related ADRs had increased since 2003 (110 ADRs), with peaks in both 2010 (470 ADRs) and 2014 (752 ADRs). In both bupropion and venlafaxine datasets, most ADRs were submitted by European Economic Area (EEA)-based pharmaceutical companies, respectively in 2,393/2,531 (94.5%) and 4,156/4,985 (83.3%) instances, with residual cases having been submitted by relevant international regulatory authorities. The bupropion drug role was judged by the reporter as “suspect” in 1,826 reports out of 2,531 (72.1%), whilst for venlafaxine the same occurred in 3,260/4,985 (65.4%) cases. For bupropion, subjects typically involved were adult males; conversely, adult females were mostly represented in venlafaxine cases. Concomitant drugs reported in bupropion cases mostly included: prescribing opiates/opioids (*n* = 123/264 cases; 46.5%); and antidepressants (*n* = 116/264; 43.9% of cases), with SSRIs and SNRIs being respectively reported in 90 and 10 cases. Concomitant drugs reported in venlafaxine cases included: prescribing opiates/opioids (*n* = 150/447 cases, 33.55%); benzodiazepines (*n* = 138/447; 30.8%); and antidepressants (*n* = 114/447; 25.5%) with SSRIs and SNRIs having been respectively reported in 70 and 18 cases. From the available data, it appeared that bupropion was administered above the therapeutic range (>300 mg/day) in seven cases, with a maximum recorded dosage of 3,000 mg. Conversely, the venlafaxine dosage was higher than the maximum typically recommended (e.g., 375 mg) in 13 cases, with the highest dosage recorded being 6,300 mg. Venlafaxine extended-release formulation was reported in 128/447 (28.6%) cases. Bupropion injecting and snorting intake practices were respectively reported in 13 and 21 cases, typically in combination with recreational drugs and/or prescribing opiates/opioids. Conversely, venlafaxine injecting and snorting intake practices were respectively reported in five and four cases, with this intake having been associated with cannabis; opiates/opioids; cocaine; and midazolam.

To better compare bupropion and venlafaxine addictive liability levels, the PRR values were computed for each ADR class (e.g., drug misuse/abuse; dependence; and withdrawal). As it appears from the following calculation, misuse/abuse ADRs appeared to be more frequently reported for bupropion than venlafaxine:

$$\begin{array}{l} \frac{\text{W}/\text{W}+\text{X}}{\text{Y}/\text{Y}+\text{Z}}=\frac{1,558/1,558+18,189}{2,361/2,361+42,531}=\frac{0.07889\;}{0.05259}=1.50 \end{array}$$

Conversely, PRR values for dependence and withdrawal resulted to be respectively 0.92 and 0.77, meaning that these ADRs were more frequently represented in venlafaxine-, as opposed to bupropion-, related reports (for all computations, see Table 2).

According to the MHRA Yellow card Scheme, the number of paroxetine, venlafaxine, fluoxetine and bupropion withdrawal-related ADRs resulted to be as follows: paroxetine: 1,358 reports out of a total number of 18,988 (7.1%); venlafaxine: 471/11,350 (4.2%); fluoxetine: 74/7,905 (0.93%); bupropion: 30/20,585 (0.14%; Table 3). In comparison with the remaining 3 ADs, bupropion was associated with the lowest values of PRR (venlafaxine vs. bupropion PRR = 29.64; paroxetine vs. bupropion PRR = 51.07), whilst venlafaxine presented with withdrawal PPR values second only to those relating to paroxetine (paroxetine vs. venlafaxine PRR: 1.72). Finally, within the SSRI group paroxetine vs. fluoxetine PRR values resulted to be of 7.61, suggesting a higher risk of withdrawal in those prescribed with paroxetine (see Table 3).

**Table 3:** Reported withdrawal adverse drug reactions for bupropion; fluoxetine; paroxetine; and venlafaxine (source: UK-based Yellow Card scheme; 2000–2016) and related PRR computations.

	**No. of reactions**	**Proportion**	**PRR computation**	
**Bupropion**		0.0014	Venlafaxine vs. Bupropion	29.64
Withdrawal reactions	30		Fluoxetine vs. Bupropion	6.71
Total reactions	20,585		Paroxetine vs. Bupropion	51.07
**Fluoxetine**		0.0094	Venlafaxine vs. Fluoxetine	4.41
Withdrawal reactions	74		Paroxetine vs. Venlafaxine	1.72
Total reactions	7,905		Paroxetine vs. Fluoxetine	7.61
**Paroxetine**		0.0715		
Withdrawal reactions	1,358			
Total reactions	18,988			
**Venlafaxine**		0.0415		
Withdrawal reactions	471			
Total reactions	11,350			

## Discussion

This study aimed at systematically identifying and analyzing venlafaxine-, as opposed to bupropion-, misuse/abuse/dependence and withdrawal issues. Present data have been extracted from a high-quality and large scale pharmacovigilance database, such as the EMA's EV that, together with the World Health Organization's Drug Monitoring Program (WHO's Drug Monitoring Programme, 2016), is considered a worldwide reference standard. Most related literature papers, so far, were based on small case series/single case studies (Quaglio et al., 2008; Vento et al., 2013). Conversely, current findings refer to much larger numbers of patients, respectively presenting with either bupropion (264 patients) or venlafaxine (447 patients) misuse/abuse/dependence and withdrawal issues. Current data and PRR values may tentatively suggest that the misuse/abuse-ADRs were more represented in bupropion cases, whilst both dependence- and withdrawal-related cases were more frequently reported for venlafaxine. Yellow Card Scheme data seemed to confirm that venlafaxine presented indeed with a higher risk of withdrawal in comparison with bupropion. Furthermore, venlafaxine withdrawal occurrence risk may be smaller, but somehow comparable, to that of paroxetine. Indeed, several papers have identified the occurrence of withdrawal signs/symptoms relating to a range of ADs, and especially SSRIs (Addictionblog.org, 2012; Harvey and Slabbert, 2014; Fava et al., 2015). The characteristics of the discontinuation syndrome have been linked to the half-life of a given SSRI, which can explain the low levels of fluoxetine discontinuation syndrome here identified (Wilson and Lader, 2015) The range of idiosyncratic (e.g., insufflation; parenteral/intravenous) intake modalities were here more frequently identified in bupropion, as opposed to venlafaxine cases. Overall, the misuse/abuse/dependence and withdrawal ADRs were here associated, for both molecules and albeit of a small proportion of cases, with supra-therapeutic, or even extraordinarily high dosages.

Although there may be no straightforward explanations for these results, the dopaminergic, stimulant-like, bupropion activities (Vento et al., 2013) may be associated with its possible recreational value. Indeed, when bupropion tablets are crushed and snorted, a high dose of the molecule is being delivered directly into the bloodstream, hence overcoming the slow-release mechanism which is typical of bupropion tablets (Renoir, 2013). Conversely, the occurrence of withdrawal phenomena after the abrupt discontinuation of venlafaxine has already been extensively described, being a real risk for each venlafaxine-treated patient (Sabljić et al., 2011). Present data focus on misuse; abuse; dependence; and withdrawal, but these are not the same issues. Although, similar to what described with the SSRIs, the occurrence of a withdrawal syndrome may be interpreted as being associated with a “dependence” condition (Fava et al., 2015), this may not necessarily be an indication, *per se*, of an index drug possessing a misuse/recreational value (Shang et al., 2007).

Apart from benzodiazepines and opiates/opioids, other ADs (mostly SSRIs) were those drugs most frequently identified in combination with both bupropion and venlafaxine. Indeed, this may suggest the comorbid presence of depression with substance misuse conditions. Unfortunately, however, the EV database did not provide here further details of clinical interest, including: possible concurrence of psychopathological conditions; medication dosage prescribed prior to discontinuation; range/intensity of withdrawal symptoms; and time-frame of the clinical presentation of withdrawal. Both bupropion and venlafaxine ADRs seemed to have increased over time. It is unclear if these trends had just mirrored the increasing rates of worldwide prescribing of these molecules. In fact, whilst English PCA data confirmed venlafaxine increase in prescribing levels in the 2009–2016 time frame, an opposite trend was identified for bupropion. Hence, one could hypothesize that the bupropion misuse increasing rates over time here identified were somehow facilitated by the progressively increasing numbers of rogue, non-prescription required, drug-vending web sites (Deluca et al., 2012; Nelson et al., 2014).

### Limitations

Some considerations are needed with respect to the dataset analyzed. Firstly, the number of case reports for a particular medicinal product may depend on its availability on the market and extent of use, as well as the public awareness of a safety concern. Moreover, the comparison here considered between venlafaxine and bupropion may be difficult, since it assumes that these drugs have similar levels of both worldwide prescription and adverse effects. Unfortunately, however, global prescribing figures are not available due to the wide differences in both availability and collection of prescription data around the world.

Case reports of suspected ADRs alone are rarely sufficient to confirm that a certain effect in a patient has been caused by a specific medicine. The fact that a suspected adverse reaction has been reported does not necessarily mean that the medicine has caused the observed effect, as this could have also been caused by the disease being treated, another illness, or it could be associated with another medicinal product taken by the patient at the same time. Also, as reports are spontaneously submitted, several ADRs relating to the same patient were here identified. This may have happened because of a range of different sources reporting the same ADR but also because a number of different ADRs may have been reported for the same patient. For this reason, report duplications may occur indeed, e.g., where a healthcare professional reported the same suspected ADR to both the national regulator and the Marketing Authorization Holder, and both eventually reported the index ADR to the EV. Finally, due to the nature of spontaneous reports, not all data fields (such as subjects' possible psychiatric/drug misuse history) were provided for all reports.

## Conclusions

Despite data collection limitations, and although further studies are clearly needed, both the literature and current EMA data may suggest that, in comparing these two molecules, bupropion appeared to be prone to misuse/abuse (Orsolini et al., 2015; Stassinos and Klein-Schwartz, 2016), whilst venlafaxine was more frequently reported as being associated with withdrawal. Present data may help the clinician in making a more informed decision about AD prescribing. One could argue, for example, that bupropion should be prescribed with caution in clients with a history of substance misuse, whilst venlafaxine and paroxetine dosage should be tapered down gradually, with the possibility of switching to fluoxetine prior to withdrawal (Wilson and Lader, 2015). Whether these abuse and withdrawal issues occur on a large scale cannot be confirmed from here but, as the EV reports were submitted spontaneously, present figures may only underestimate the magnitude of the problem.

The reasons for non-medical use of prescription drugs are complex. However, a range of factors may well facilitate this occurrence, including: perception of prescription drugs' non-medical use as being more socially acceptable, less stigmatized, and safer (Hu et al., 2016) than the intake of scheduled/illicit substances; and likely lack of detection in standard drug screens.

Healthcare professionals should be vigilant when prescribing any psychotropics, including ADs (Carvalho et al., 2016), and particularly so to inmates and/or those with a substance misuse history. The amount of drug prescribed per individual prescription should be limited; and, if any related misuse issues are being identified, physicians should consider medication tapering (Evans and Sullivan, 2014).

## Author contributions

Both FS and SC conceived the idea of the manuscript, analyzed the data here presented and drafted the manuscript. Final responsibility of manuscript content remains with FS.

### Conflict of interest statement

The authors declare that the research was conducted in the absence of any commercial or financial relationships that could be construed as a potential conflict of interest.
